# A tail of two pandas— whole genome k-mer signature analysis of the red panda (*Ailurus fulgens*) and the Giant panda (*Ailuropoda melanoleuca*)

**DOI:** 10.1186/s12864-021-07531-3

**Published:** 2021-04-01

**Authors:** Matyas Cserhati

**Affiliations:** Independent Scholar, 2615C Muscatel Avenue, Rosemead, CA 91770 USA

**Keywords:** Red panda, Giant panda, Whole genome k-mer signature, Pearson correlation, Mustelid, Ursid, Procyonid, Mephitid

## Abstract

**Background:**

The red panda (*Ailurus fulgens*) is a riddle of morphology, making it hard to tell whether it is an ursid, a procyonid, a mustelid, or a member of its own family. Previous genetic studies have given quite contradictory results as to its phylogenetic placement.

**Results:**

A recently developed whole genome-based algorithm, the Whole Genome K-mer Signature algorithm was used to analyze the genomes of 28 species of Carnivora, including *A. fulgens* and several felid, ursid, mustelid, one mephitid species. This algorithm has the advantage of holistically using all the information in the genomes of these species. Being a genomics-based algorithm, it also reduces stochastic error to a minimum. Besides the whole genome, the mitochondrial DNA from 52 mustelids, mephitids, ursids, procyonids and *A. fulgens* were aligned to draw further phylogenetic inferences.

The results from the whole genome study suggested that *A. fulgens* is a member of the mustelid clade (*p* = 9·10^− 97^). *A. fulgens* also separates from the mephitid *Spilogala gracilis*. The giant panda, *Ailuropoda melanoleuca* also clusters away from *A. fulgens*, together with other ursids (*p* = 1.2·10^− 62^). This could be due to the geographic isolation of *A. fulgens* from other mustelid species. However, results from the mitochondrial study as well as neighbor-joining methods based on the sequence identity matrix suggests that *A. fulgens* forms a monophyletic group. A Maximum Likelihood tree suggests that *A. fulgens* and Ursidae form a monophyletic group, although the bootstrap value is weak.

**Conclusions:**

The main conclusion that we can draw from this study is that on a whole genome level *A. fulgens* possibly belongs to the mustelid clade, and not an ursid or a mephitid. This despite the fact that previously some researchers classified *A. fulgens* and *A. melanoleuca* as relatives. Since the genotype determines the phenotype, molecular-based classification takes precedence over morphological classifications. This affirms the results of some previous studies, which studied smaller portions of the genome. However, mitochondrial analyses based on neighbor-joining and maximum likelihood methods suggest otherwise.

**Supplementary Information:**

The online version contains supplementary material available at 10.1186/s12864-021-07531-3.

## Background

The red panda (*Ailurus fulgens*) is an enigmatic animal and is hard to classify based on its morphology. It lives in parts of India, Nepal and China, and has a distinct red-white coloration, and a striped, bushy tail. It goes by several nicknames, such as the ‘bear-cat’, the ‘cat-bear’, the ‘lesser panda’ or the ‘fire-fox’. Some researchers think *A. fulgens* is a relative of the giant panda (*Ailuropoda melanoleuca*) based on several physical characteristics. These include an almost exclusive diet of bamboo (both species eat meat on occasion), and have an enlarged radial sesamoid bone, which they use to process bamboo [1, 2].

Because of these similarities, the giant panda even received its name from the red panda. According to other opinions, *A. fulgens* has been classified as a member of the family Procyonidae (raccoons). Yet others put the red panda into its own family (Ailuridae) [3]. *A. fulgens* also has some unique characteristics: a large zygomatic arch, a powerful jaw, and complex cheek teeth, following a P2–3 pattern [1].

According to new genetic evidence, there are two species of red panda, the Himalayan red panda (*A. fulgens*), and the Chinese red panda (*A. styani*) [4]. Due to reduced numbers, the red panda is an endangered species. Previous studies based on different combinations of nuclear and mitochondrial genes have given contradictory results as to the taxonomic relationship of *A. fulgens* with other carnivores. This may be because only several mitochondrial and/or nuclear genes were analyzed, and not the entire whole genome sequence (WGS).

The red panda’s classification as a procyonid or procyonid-relative is based on immunological, DNA-DNA hybridization, and isozyme evidence [5]. A phylogenetic tree based on Bayesian analysis of cytochrome-b put *A. fulgens* next to Canidae [6].

For example, Peng et al. classify *A. fulgens* either as a mustelid, placing them next to the American marten (*Martes americana*), or as a mephitid, next to the striped skunk (*Mephitis mephitis*). This was based on the analysis of 13 concatenated mitochondrial proteins, based on neighbor-joining (NJ) and maximum likelihood (ML) phylogenetic methods, respectively [7]. In a study of three mtDNA genes (12S rRNA, 16S rRNA and cytochrome b) and intron 1 of the nuclear transthyretin gene, Flynn et al. also found that *A. fulgens* is neither an ursid, nor a procyonid, nor a mephitid, but a mustelid [1]. Another study including three mitochondrial and three nuclear genes by Fulton and Strobeck, based on 16 arctoid species, with *Canis lupus* as an outlier, placed *A. fulgens* in close relationship to *M. mephitis* [8].

Yu and Zhang studied introns 4 and 7 from the nuclear gene ß-fibrinogen (FGB) as well as the mitochondrial gene NADH dehydrogenase subunit 2 (ND2) in 17 species from the order Carnivora. In their results, these researchers found that *A. fulgens* is most closely related to procyonids based on analysis of intron 4 of the FGB gene. But when intron 7 was analyzed, it clustered towards ursids. Classification based on the ND2 gene *A. fulgens* clustered with mustelids, but these results had poor bootstrapping support. When the two introns were combined with analysis of the genes IRBP and TTR, *A. fulgens* was closest to mustelids [9].

Sato et al. analyzed a 5.5 Kbp segment of DNA coding for five genes, AOPB, BRCA1, RAG1, RBP3, and VWF, and found that *A. fulgens* clusters together with procyonids and mustelids, and not with mephitids (skunks and stink badgers) [10]. An earlier, similar result was attained when studying a 3.2 Kbp segment containing the genes APOB, RAG1 and IRBP [11]. Genomically, *A. fulgens* shares several apomorphic chromosome fusions with mustelids, namely F2 + C1p and A1p + C1q [12]. However, *A. fulgens* differs in several other chromosomal rearrangements indicating that it diverged early from other mustelids.

Interestingly, several genes have been found in both species which show convergent development. For example, changes in the amino acid composition of the DYNC2H1 and PCNT proteins lead to polydactyly in humans and mice, but to the pseudo-thumb in the giant and red pandas. Three other convergent genes (PRSS1, PRSS36, and CPB1) are responsible for more efficient uptake of nutrients from bamboo, which makes up a large part of their diet as well. Four other genes, ADH1C, CYP3A5, CYP4F2, and GIF also enable the more effective utilization in the giant and red pandas of vitamins A and B12 as well as arachidonic acid, which are absent or very low in bamboo [2].

Intron analysis is useful, since these sequences are not under selection pressure. An analysis of 22 Kbp of nuclear intron sequences from 16 carnivore species groups *A. fulgens* with Musteloidea sensu stricto (Mustelidae+Procyonidae) to the exclusion of mephitids [13]. These results, however, contradict results coming from mtDNA analyses [14].

Since morphology-based classification of *A. fulgens* is ambiguous, it would be helpful to determine the precise taxonomic status of this species based on a whole genome-based algorithm. To this end, the Whole Genome K-mer Signature (WGKS) algorithm [15] is used to analyze the genomes of five bear species, eleven cat species and ten species from the family Mustelidae (weasels, otters, martens, and badgers), *Spilogala gracilis*, a mephitid species, as well as *A. fulgens*, making 28 species in total.

The advantages of using a genomics-based algorithm to analyze the WGS of these organisms is that it takes all the information present in the WGS, as opposed to just a handful of genes, utilized in gene studies. Deciding which genes are important is subjective and may vary between investigators. Whole genome-based algorithms also have the advantage that they greatly reduce stochastic error, due to the vast number of characters (DNA bases) that they analyze [16]. Using this algorithm can provide additive results as to the phylogenetic classification of *A. fulgens*.

While the WGKS algorithm may not be a sensu stricto phylogenetic algorithm, it can still be used to classify species, based on their WGS into different groups. There are several metagenomics methods that use k-mer analysis to map Next-Generation read sequences to species represented by whole genome sequences, such as kraken [17], the Naïve Bayes Classifier (NBC) [18], and PhymmBL [19]. For example, kraken splits read sequences into k-mers, which it then maps to a taxonomic tree. The leaf node/species which has the most reads assigned to it is the designated as the species that the read came from. The NBC also splits a read into constituent N-mers, and then calculates the a posteriori probability of a given N-mer belonging to a specific strain, species, genus, or other taxon.

The NBC algorithm and the WGKS algorithm are similar in that they both utilize the k-mer signature of a DNA sequence in order to classify it. One could view the whole genome sequence as a very extended read sequence. Using k-mer methods on whole genome sequences (WGS) should give even more accurate results than on read sequences because the WGS represents a much larger search space. Individual k-mers occur in much larger numbers than in short reads, which are between 75 and 300 bp or so. In other words, the k-mer ‘coverage’ is much, much higher in a WGS than a single read.

Besides a whole genome approach, it would also be useful to complement the results from the whole genome analysis using a multiple alignment of several genes. To this end the mitochondrial DNA of 52 ursid, mephitid, mustelid, procyonid species along with the ailuronids, *A. fulgens* and *A. fulgens styani* were analyzed. Not only does the mtDNA contain more than a dozen conserved genes, these genes are localized to the same part of the genome and also largely follow the same order. The mtDNA also contains non-coding DNA, which is not under selection pressure, and thus better reflects species relationships. Mitochondrial genes would be more conducive to this kind of analysis as opposed to artificially concatenating together genes from different parts of the genome. These mtDNA sequences were aligned using the online MUSCLE tool at the EBI website. Species relationships were also examined using the Neighbor-joining (NJ) method as well as the Maximum Likelihood (ML) method using bootstrap values.

## Results and discussion

### Pre-clustering analysis of WGS

The list of species used in this analysis, the resulting PCC matrix, clusters and statistics can be seen in Additional File 1 online. The Hopkins statistic is 0.9, which means that the data set is of very good quality for clustering. The silhouette plot (Supplementary figures 1 and 2) gave a maximum average silhouette width of 0.82 for three clusters and 0.8 for four clusters. The average silhouette width was studied for two to seven clusters. The only difference was the placement of the mephitid, *S. gracilis* into its own group (cluster 4 in Supplemental figure 2).

### Whole genome analysis

**Fig. 1:**
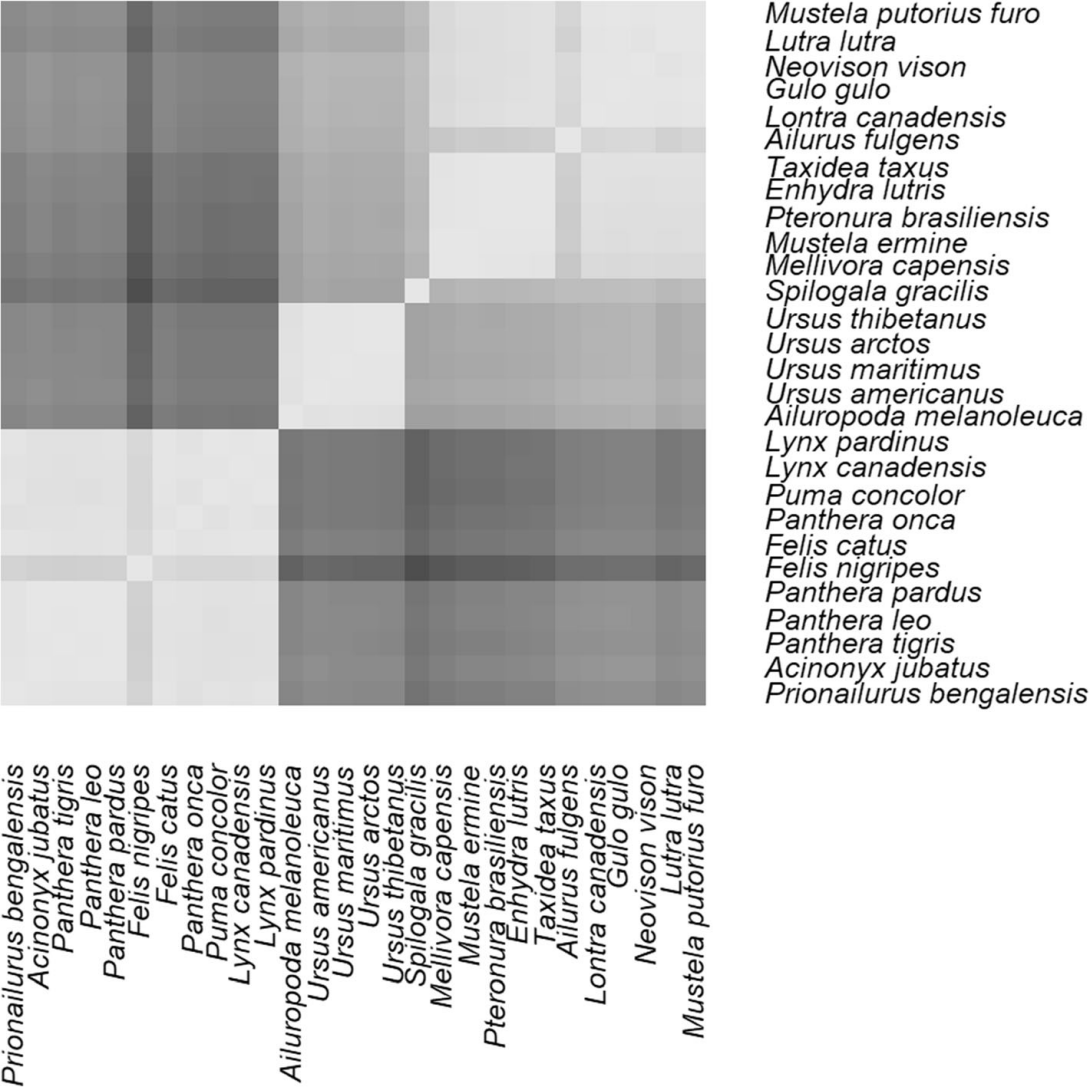
Heatmap depicting group relationships for 28 species based on results from the WGKS algorithm. Brighter colors represent species pairs which are in the same group, with a PCC value closer to 1. Darker colors represent species pairs which are in different group, with a PCC less than 1

In Fig. 1 we can see three visible clusters, felids, ursids and mustelids, with *S. gracilis* in between the mustelids and the ursids. Based on the results in Table 1, *A. fulgens* clearly clusters together with the mustelids, although on average, it has a lower mean PCC value compared to all the other species, 0.89 ± 0.03, whereas mustelids have a mean PCC value of 0.95 ± 0.04.

**Table 1:** Classification of the 28 species used in the WGKS analysis

Species	Group
*Ailurus fulgens*	1
*Enhydra lutris*	1
*Gulo gulo*	1
*Lontra canadensis*	1
*Lutra lutra*	1
*Mellivora capensis*	1
*Mustela ermine*	1
*Mustela putorius furo*	1
*Neovison vison*	1
*Pteronura brasiliensis*	1
*Taxidea taxus*	1
*Ailuropoda melanoleuca*	2
*Ursus americanus*	2
*Ursus arctos*	2
*Ursus maritimus*	2
*Ursus thibetanus*	2
*Acinonyx jubatus*	3
*Felis catus*	3
*Felis nigripes*	3
*Lynx canadensis*	3
*Lynx pardinus*	3
*Panthera leo*	3
*Panthera onca*	3
*Panthera pardus*	3
*Panthera tigris*	3
*Prionailurus bengalensis*	3
*Puma concolor*	3
*Spilogala gracilis*	4

This difference is not too significant. If we compare *Felis nigripes* (the black-footed cat) with other cats, it has a mean PCC value of 0.89 ± 0.02, whereas felids having an even greater mean PCC of 0.97 ± 0.03. Yet we know that cats are a monophyletic group. Table 2 shows the minimum, mean, maximum PCC for all three putative clades, as well as the *p*-value, which is statistically significant for all three groups.

**Table 2:** Statistical measures for each of the three clusters in the WGKS analysis

Group	Name	No. species	Min	Mean	Max	stdev	***P***-value
1	mustelids	11	0.841	0.954	0.999	0.04	8.97E-97
2	ursids	5	0.966	0.983	0.997	0.012	1.23E-62
3	felids	11	0.879	0.965	0.998	0.032	6.17E-95

**Fig. 2:**
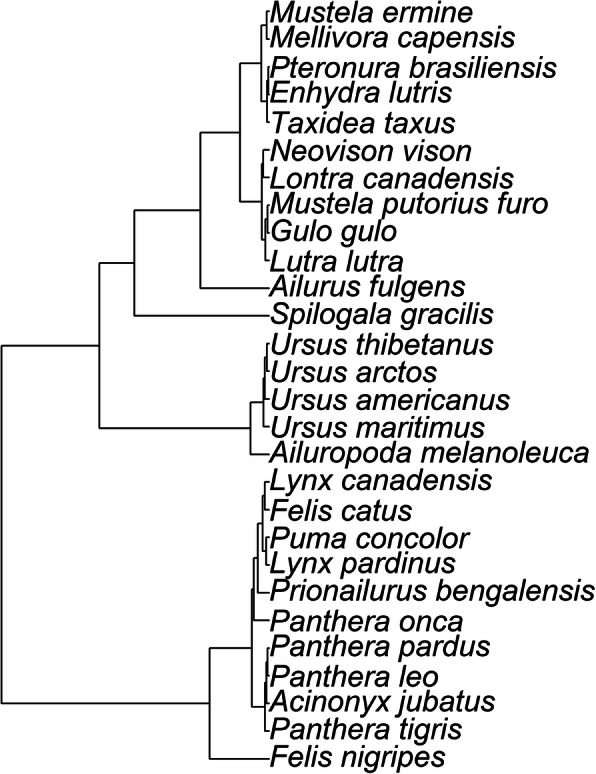
UPGMA-based hierarchical tree for the 28 species based on PCC values. Ursids, felids, and mustelids form separate clades, with *S. gracilis* in its own group

Based on this evidence, *A. fulgens* would belong to mustelids as a monophyletic group. Since it has such a low mean PCC is because it may have diverged early from other mustelids, possibly due to its isolated mountainous habitat in parts of Myanmar, Burma and China. This can also be seen well in Fig. 2, which shows the UPGMA-based phylogenetic tree for the 28 species in the whole genome analysis.

Also important is that the skunk species *S. gracilis* does not cluster with mustelids. When compared with mustelids, *S. gracilis* has a mean PCC value of 0.78 ± 0.02. *A. fulgens* has a PCC value of 0.79 with this species as opposed to a mean PCC value of 0.89 with mustelids, reported previously. This also indicates that mustelids and mephitids form separate clades.

The giant panda, *Ailuropoda melanoleuca* is a clearly a member of a clade which includes the ursids, as shown in Fig. 2. It has a mean PCC value of 0.97 ± 0.003 with the other ursids. Other genetic evidence classifies the giant panda as a member of Ursidae. This includes mtDNA, chromosome banding patterns, and serological and immunological evidence [20, 21].

### Analysis of mitochondrial genomes

**Fig. 3:**
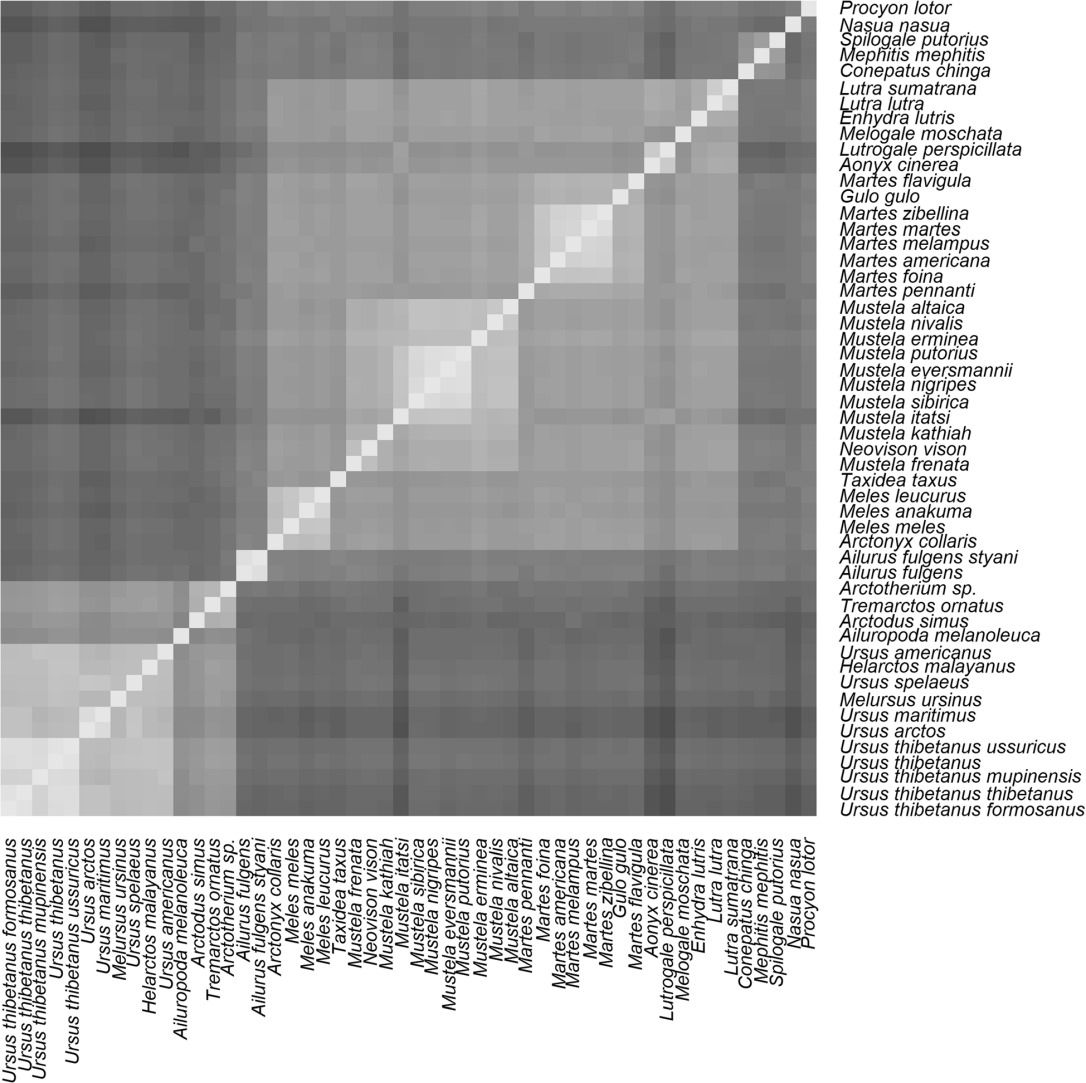
Heatmap depicting group relationships for 52 carnivore species based on alignment of the mitochondrial genome using the online MUSCLE software. Brighter colors represent species pairs which are in the same group, with a sequence identity closer to 1. Darker colors represent species pairs which are in different group, with a sequence identity closer to 0

The result of the analysis of the mitochondrial genomes can be seen in Fig. 3. The Hopkins clustering statistic is 0.841, which means that the sequence identity matrix is of good quality for clustering. Three larger clusters and two smaller clusters are visible in the heat map. The clusters and statistics for these five groups are available in the ‘clusters’ and ‘stats’ tab of Additional File 2, and Table 3 respectively. The list of species, accession numbers, and the results from this analysis are also available online at github in Additional File 2.

**Table 3:** Statistical measures for each of the five clusters in the mitochondrial analysis

Group	Name	No. species	Min	Mean	Max	stdev	*P*-value
**Ursidae**							
1	ursids	15	0.811	0.880	0.989	0.048	5.0E-41
**Musteloidea**							
2–5	Musteloidea	37	0.837	0.769	0.837	0.981	3.3E-185
2	mustelids	30	0.822	0.858	0.981	0.029	1.7E-201
3	ailuronids	2	0.980	0.980	0.980	NA	2.0E-122
4	procyonids	2	0.803	0.803	0.803	NA	1.9E-17
5	mephitids	3	0.830	0.838	0.849	0.01	0.012

**Fig. 4:**
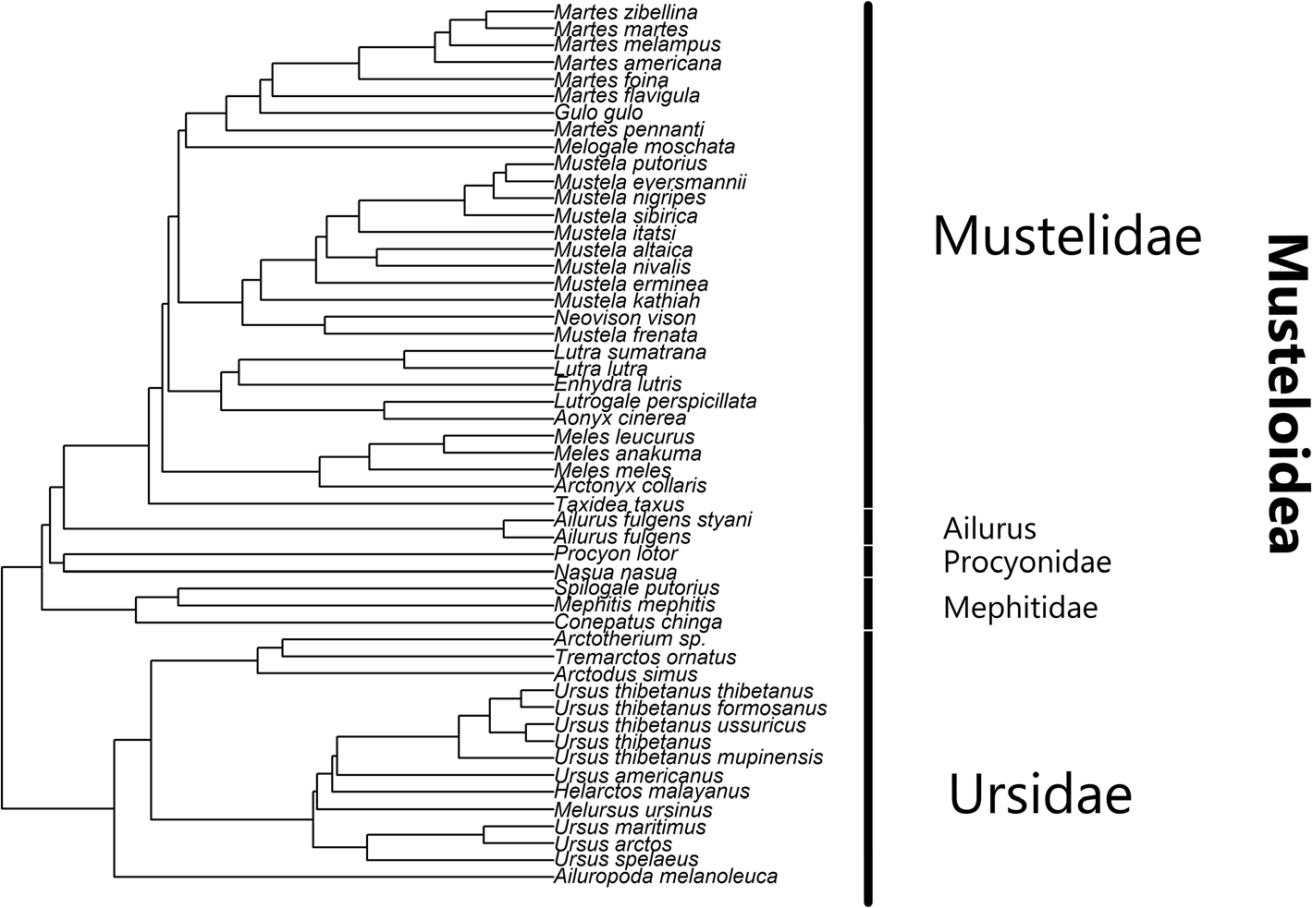
UPGMA-based hierarchical tree for the 52 species analyzed in the mtDNA study, based on sequence identity metrics. Mustelids and ursids form two large clades, and mephitids, procyonids forming two small groups. *A. fulgens* and *A. fulgens styani* appear either to form their own clade, or loosely associate with mustelids

Figure 4 depicts a hierarchical tree, showing the position of the different clades. Ursids and Musteloidea form two large clades, with 15 and 37 species, respectively. Within Musteloidea we have three smaller groups besides Mustelidae. The first one consists of both species of *A. fulgens.* The second is made up of three mephitids, *S. gracilis*, *M. mephitis*, and *Conepatus chinga*. Lastly, two procyonids, *Procyon lotor* (raccoon), and *Nasua nasua* (ring-tailed coati) make up the third group. Supplementary figure 3 shows the average silhouette width according to the number of clusters, with an average silhouette width of 0.51 for two clusters.

**Fig. 5:**
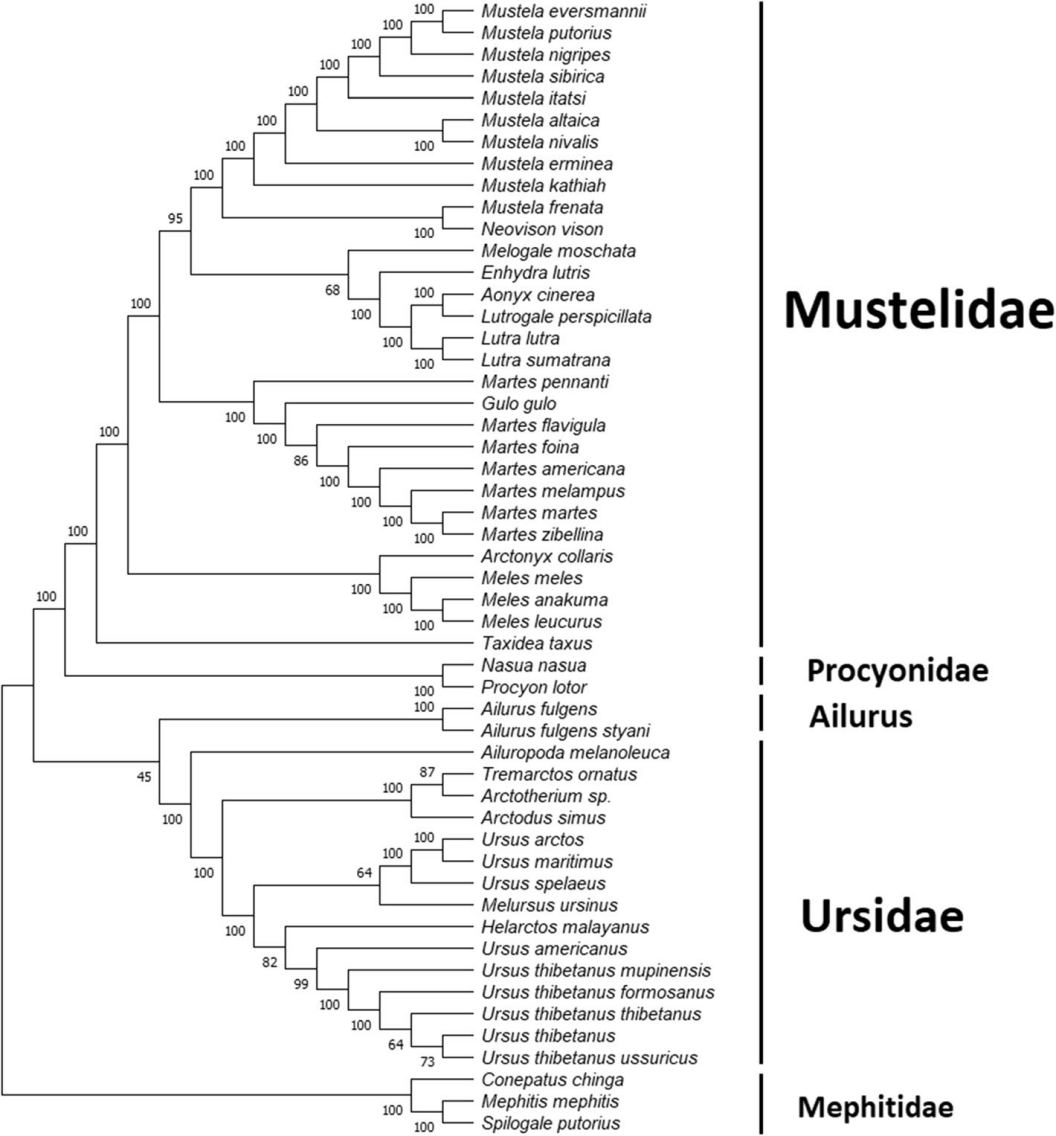
Hierarchical tree constructed using the Neighbor-Joining method using the MEGA-X software. The bootstrap consensus tree was inferred from 1000 replicates. Branches corresponding to partitions reproduced in less than 50% bootstrap replicates are collapsed. The percentage of replicate trees in which the associated taxa clustered together in the bootstrap test are shown next to the branches. This analysis involved 52 nucleotide sequences, with a total of 17,440 positions in the final dataset

Figure 5 shows the hierarchical tree constructed using the NJ method. Mustelidae forms a well-defined clade, with almost all branch points supported with a bootstrap value of 100. *N. nasua* and *P. lotor* form a smaller clade right next to Mustelidae. The three mephitids, *C. chinga*, *M. mephitis* and *S. putorius* also form a small clade, well separated from the other clades. The NJ method places *Ailurus* next to Ursidae, suggesting that they possibly form a monophyletic group. However, the node connecting *Ailurus* with Ursidae only has a bootstrap value of 45.

**Fig. 6:**
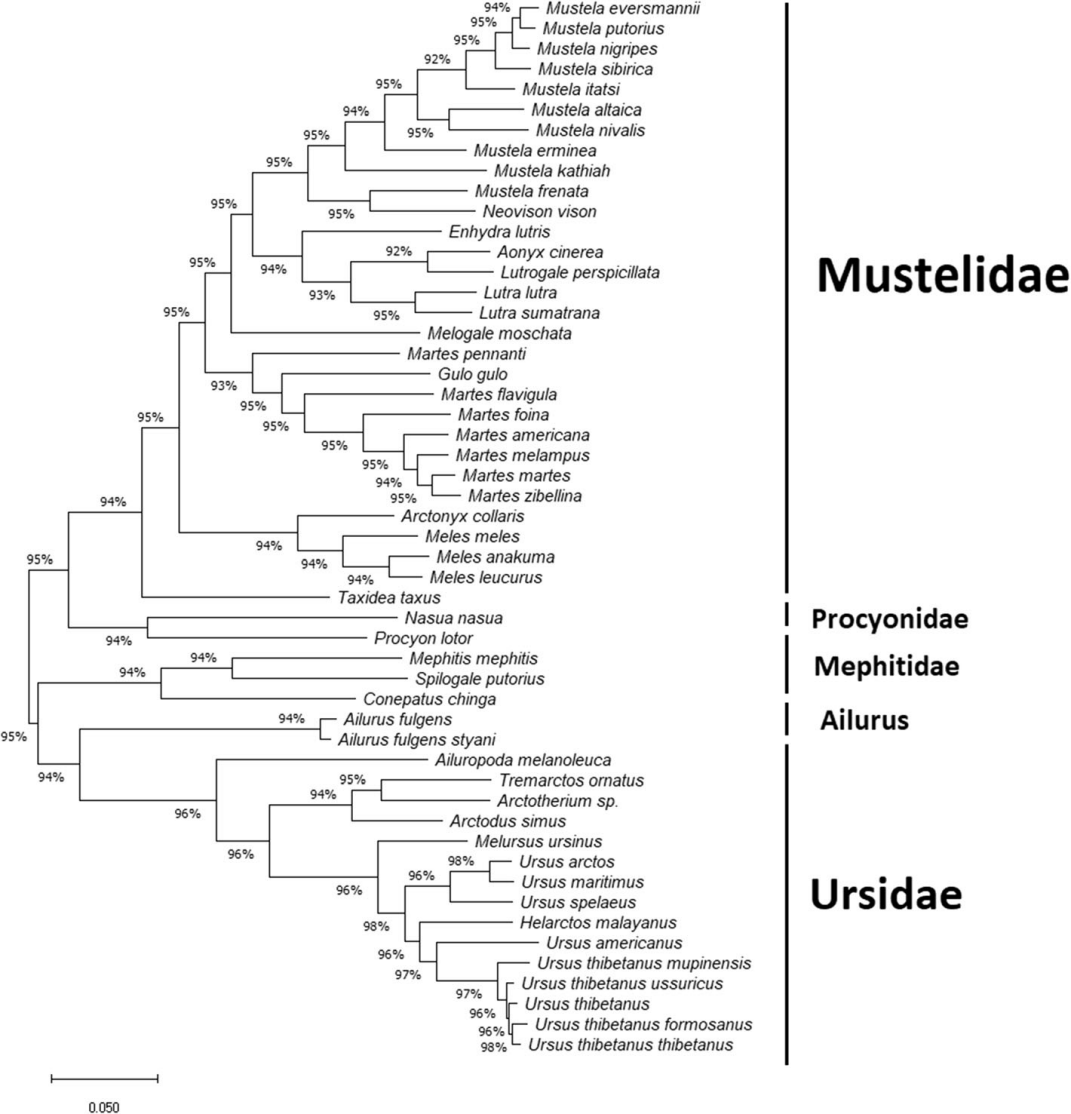
Hierarchical tree inferred using the Maximum Likelihood method and Tamura-Nei model using the MEGA-X software. The tree with the highest log likelihood (− 251,658.67) is shown. Initial tree(s) for the heuristic search were obtained automatically by applying Neighbor-Join and BioNJ algorithms to a matrix of pairwise distances estimated using the Tamura-Nei model, and then selecting the topology with superior log likelihood value. The tree is drawn to scale, with branch lengths measured in the number of substitutions per site. The proportion of sites where at least one unambiguous base is present in at least one sequence for each descendent clade is shown next to each internal node in the tree. This analysis involved 52 nucleotide sequences, with a total of 17,440 positions in the final dataset

Figure 6 show the hierarchical tree constructed using the ML method. Here Mustelide, Procyonidae, and Mephitidae all form their own clades with a likelihood value of at least 94%. As opposed to the NJ tree, here *Ailurus* is separated from Ursidae suggesting that it might form its own clade as well. Ledje et al. [3] also found that *A. fulgens* was distinct from all other caniforms, and placed it in its own monotypic family. However, this analysis was based on the analysis of only the mitochondrial 12S rRNA gene. Flynn et al. also reached a similar conclusion based on the analysis of three mitochondrial genes [1]. On the other hand, Peng et al. [7] classified *A. fulgens* as a mustelid, based on the analysis of thirteen concatenated mitochondrial proteins.

These results may seem to contradict the results of the WGKS analysis, by placing *A. fulgens* into a monophyletic group, separated from mustelids. Let us bear in mind, that even though the mitochondrial genome is a good way to study multi-gene alignments, it is still only a fraction of the entire genome. We must also remember that *A. fulgens* is a geographically isolated species, which may lead to its genetic isolation from other mustelids as well. Fulton and Strobeck analyzed four nuclear sequence-tagged sites and one exon of the gene IRBP within 79 carnivore species, and also found discordant results between the results of the mitochondrial and nuclear analyses. In their study, the mtDNA results supported the monophyly of Ailuridae and Mephitidae, whereas the nuclear results suggested otherwise [22].

## Conclusion

In conclusion, *A. fulgens* possibly belongs to Mustelidae, based on the analysis of the WGKS. This species also clusters away from *S. gracilis*, indicating that mustelids and mephitids belong to separate clades, which is reinforced by the mtDNA results as well. This is based on whole genome data as opposed to the contradictory results in previous studies involving just a handful of genes, one even in two different exons of the same gene. This demonstrates the utility of the WGKS algorithm, which takes a holistic approach of analyzing the WGS.

The mtDNA results as well as the maximum likelihood tree appear to place *A. fulgens* into a monophyletic group. *A. melanoleuca*, on the other hand, belongs to the ursids, as shown consistently in both the WGS results as well as the mtDNA results and the NJ and ML trees. Based on neighbor-joining methods, it appears that *Ailurus* could form a monophyletic group with ursids, but the bootstrap value is too low to say this with certainty.

## Methods

### Data and programs used

The Python script motif_analysis_k-1.py at github.com/csmatyi/motif_analysis was used to generate WGKS profiles. Version 3.6.0. of R was used. The heatmap was generated using the R command ‘heatmap’, using the ‘ward. D’ clustering algorithm for the WGKS analysis, and the ‘single’ algorithm for the mitochondrial data. Clusters were generated using the ‘cutree’ command and were depicted in hierarchical trees using the UPGMA method [23]. To determine the optimal number of clusters, the ‘cluster’ and ‘factoextra’ libraries and the fviz_nbclust command were used, setting the method parameter to ‘wss’. The ‘fviz_silhouette’ plot was used to construct the Silhouette plot. The WGS of the 28 species used in the WGKS analysis and the 52 complete mitochondrial genome Refseq sequences were downloaded from the nucleotide database at NCBI. Additional Excel files and figures as well as the mitochondrial genome fasta file can be found online at github.com/csmatyi/ailurus.

### Description of algorithm

The WGKS algorithm that was used in the analysis is an alignment-free k-mer sequence comparison method [24]. These methods involve the statistical comparison of k-mers between species. A k-mer is a segment of DNA k bp long, which can correspond to the core segment of a transcription factor binding site, a repeat element or other regulatory element. These elements take part in protein binding and gene regulation and are conserved across different species. The advantages of using a k-mer based alignment-free algorithms over alignment-based ones is that they process input much faster and are unbiased by guide trees imposed upon the data [25, 26].

For a lengthy description of the algorithm, the reader is referred to Cserhati et al. [15]. However, a short description is provided here for better understanding. The WGKS algorithm is divided into three steps.

First, all possible k-2, k-1, and k-mers in the genome of a given species are enumerated to give the observed occurrence O. Then, based on these observed occurrences, the expected occurrence E can also be calculated with the following equation:

1 $$ {\mathrm{E}}_{\mathrm{k}}={\mathrm{O}}_{1..\mathrm{k}-1}\bullet {\mathrm{O}}_{2..\mathrm{k}}/{\mathrm{O}}_{2..\mathrm{k}-1} $$

where E_k_ is the expected occurrence of the k-mer, O_1..k-1_ is the observed occurrence of the k-1-mer from positions 1 to k-1, O_2..k_ is the observed occurrence of the k-1-mer from positions 2 to k, and O_2..k-1_ is the observed occurrence of the k-2-mer from positions 2 to k-1.

The score value S can be calculated in the following way:

2 $$ {S}_{k- mer}=\frac{O-E}{O+E} $$

Score values can be interpreted in three ways:

3 $$ O\gg E:{S}_{k- mer}\to 1\kern0.5em \left(\mathrm{overrepresnted}\kern0.5em \mathrm{k}\hbox{-} \mathrm{mer}\right) $$

4 $$ O\ll \kern0.5em E:\kern0.5em {S}_{k- mer}\to -1\kern0.5em \left(\mathrm{underrepresented}\kern0.5em \mathrm{k}\hbox{-} \mathrm{mer}\right) $$

5 $$ O=\kern0.5em E:{S}_{k- mer\kern0.5em }\approx 0\kern0.5em \left(\mathrm{ramdomly}\kern0.5em \mathrm{occurring}\kern0.5em \mathrm{k}\hbox{-} \mathrm{mer}\right) $$

Even if the genome is partially or completely duplicated, then the score value will not change. This is because both the Observed and Expected values will increase by the proportion that the duplicated genome is compared to the pre-duplication genome.

The next step involves comparing the k-mer signature between two species. The k-mer signature is simply a list of all k-mers ordered in lexicographical order from AA … A to TT … T, together with their score values. For a given value k, there are 4^k^ possible k-mers. Thus, the k-mer signature also corresponds to a vector of 4^k^ numbers. Since octamers were analyzed, this corresponds to 65,536 possible octamers. Two of these vectors can be compared to one another for two different species using the Pearson Correlation Coefficient (PCC). PCC values closer to 1 represent a pair of closely related species, within the same clade. Lower PCC values denote two unrelated species. This step is performed between all possible pairs of species to derive a square PCC matrix. *P*-values for clusters were calculated by comparing the PCC values for all species pairs within the cluster with all PCC values for all species pairs where one species came from the cluster, and the other species was outside the cluster.

The last step involves visualizing the PCC in a heatmap and using clustering algorithms to detect monophyletic groups. Clustering can be done for example using the k-means clustering algorithm, or the Partitioning Among Medoids (PAM) algorithm.

### Construction of hierarchical trees

Hierarchical trees were drawn with the Neighbour Joining [27] and Maximum Likelihood [28] methodologies, using bootstrap values. Both trees were constructed using the MEGA-X software [29], with parameters set to default values. For the NJ method, the Maximum Composite Likelihood model was used. 1000 bootstrap replications were used for the construction of both trees. For the ML method, the Tamura-Nei model was used with uniform rates.

### Mitochondrial DNA analysis

The 52 complete mitochondrial genome sequences for the ursid, mephitid, mustelid, procyonid species and the two *A. fulgens* species were aligned using the online MUSCLE tool [30], version 3.8 at ebi.ac.uk/Tools/msa/muscle using default parameters. The sequence identity matrix was derived from the alignment using BioEdit, version 7.2.5 [31].

## Supplementary Information

**Additional file 1.** Results of whole genome analysis of 28 species. The file includes a list of species, and the genome sequence files downloaded from NCBI, the PCC matrix which is a result of the WGKS algorithm, as well as the species clusters and the cluster statistics.

**Additional file 2.** Results of the alignment of mitochondrial DNA from 52 carnivore species. This file includes a list of species and their corresponding accession number, the sequence identity matrix, species clustering information and cluster statistics.

**Additional file 3: Figure S1.** Silhouette plot for three clusters from the WGKS analysis. The average silhouette width is 0.82.

**Additional file 4: Figure S2.** Silhouette plot for four clusters from the WGKS analysis. The average silhouette width is 0.8.

**Additional file 5: Figure S3.** Plot showing the mean silhouette width according to the number of clusters for the mitochondrial data, based on the ‘silhouette’ method. The maximum average silhouette width is 0.51 for two clusters.

## Data Availability

The Python script motif_analysis_k-1.py at github.com/csmatyi/motif_analysis was used to generate WGKS profiles. Additional files and figures can be found online at github.com/csmatyi/ailurus. Accession numbers of the 28 WGS from the National Center for Biotechnology Information (NCBI): **Table:** 

*Acinonyx jubatus*	GCF_003709585.1
*Felis catus*	GCF_000181335.3
*Felis nigripes*	GCA_004023925.1
*Lynx canadensis*	GCF_007474595.1
*Lynx pardinus*	GCA_900661375.1
*Panthera leo*	GCA_008795835.1
*Panthera onca*	GCA_004023805.1
*Panthera pardus*	GCF_001857705.1
*Panthera tigris*	GCF_000464555.1
*Prionailurus bengalensis*	GCA_005406085.1
*Puma concolor*	GCF_003327715.1
*Spilogale gracilis*	GCA_004023965.1
*Enhydra lutris*	GCF_002288905.1
*Gulo gulo*	GCA_900006375.2
*Lontra canadensis*	GCA_900006375.2
*Lutra lutra*	GCA_902655055.1
*Mellivora capensis*	GCA_004024625.1
*Mustela erminea*	GCF_009829155.1
*Mustela putorius furo*	GCF_000215625.1
*Neovison vison*	GCA_900108605.1
*Pteronura brasiliensis*	GCA_004024605.1
*Taxidea taxus*	GCA_003697995.1
*Ailurus fulgens*	GCA_002007465.1
*Ailuropoda melanoleuca*	GCF_000004335.2
*Ursus americanus*	GCA_003344425.1
*Ursus arctos*	GCF_003584765.1
*Ursus maritimus*	GCF_000687225.1
*Ursus thibetanus*	GCA_009660055.1 Accession numbers of 52 mtDNA sequences from the National Center for Biotechnology Information: **Table:** 

*Ailuropoda melanoleuca*	NC_009492.1
*Ailurus fulgens*	NC_011124.1
*Ailurus fulgens styani*	NC_009691.1
*Aonyx cinerea isolate 16*	NC_035814.1
*Arctodus simus*	NC_011116.1
*Arctonyx collaris voucher YP6001*	NC_020645.1
*Arctotherium sp.*	NC_030174.1
*Conepatus chinga*	NC_042596.1
*Enhydra lutris*	NC_009692.1
*Gulo gulo*	NC_009685.1
*Helarctos malayanus*	NC_009968.1
*Lutra lutra*	NC_011358.1
*Lutra sumatrana isolate 49*	NC_035810.1
*Lutrogale perspicillata isolate 21*	NC_035811.1
*Martes americana voucher ROM116315*	NC_020642.1
*Martes flavigula*	NC_012141.1
*Martes foina voucher YP6135*	NC_020643.1
*Martes martes*	NC_021749.1
*Martes melampus*	NC_009678.1
*Martes pennanti isolate MP41*	NC_020664.1
*Martes zibellina*	NC_011579.1
*Meles anakuma*	NC_009677.1
*Meles leucurus*	NC_039173.1
*Meles meles*	NC_011125.1
*Melogale moschata voucher YP6128*	NC_020644.1
*Melursus ursinus*	NC_009970.1
*Mephitis mephitis voucher YP2994*	NC_020648.1
*Mustela altaica*	NC_021751.1
*Mustela erminea*	NC_025516.1
*Mustela eversmannii*	NC_028013.1
*Mustela frenata voucher ROM101452*	NC_020640.1
*Mustela itatsi isolate IT01*	NC_034330.1
*Mustela kathiah voucher YP6126*	NC_023210.1
*Mustela nigripes*	NC_024942.1
*Mustela nivalis voucher YP1*	NC_020639.1
*Mustela putorius voucher ROM117143*	NC_020638.1
*Mustela sibirica voucher YP6136*	NC_020637.1
*Nasua nasua voucher ROM108237*	NC_020647.1
*Neovison vison voucher ROM102488*	NC_020641.1
*Procyon lotor*	NC_009126.1
*Spilogale putorius*	NC_010497.1
*Taxidea taxus voucher ROM111450*	NC_020646.1
*Tremarctos ornatus*	NC_009969.1
*Ursus americanus*	NC_003426.1
*Ursus arctos*	NC_003427.1
*Ursus maritimus*	NC_003428.1
*Ursus spelaeus*	NC_011112.1
*Ursus thibetanus*	NC_009971.1
*Ursus thibetanus formosanus*	NC_009331.1
*Ursus thibetanus mupinensis*	NC_008753.1
*Ursus thibetanus thibetanus*	NC_011118.1
*Ursus thibetanus ussuricus*	NC_011117.1

## Abbreviations

Ailuronid: A member of the family Ailuronidae, the red panda
Mephitid: A member of the family Mephitidae, or skunks
ML: Maximum Likelihood method
mtDNA: Mitochondrial DNA
Mustelid: A member of the family Mustelidae, or a group of animals including weasels, otters, ferrets, minks, martens and wolverines.
NJ: Neighbor Joining method
PCC: Pearson Correlation Coefficient
Procyonid: A member of the family Procyonidae, or raccoons
UPGMA: Unweighted pair group method with arithmetic mean, am agglomerative hierarchical clustering method
Ursid: A member of the family Ursidae, or bears
WGS: Whole genome sequence
WGKS: Whole genome k-mer signature

## References

[1] Flynn JJ, Nedbal MA, Dragoo JW, Honeycutt RL (2000). Whence the red panda?. Mol Phylogenet Evol.

[2] Hu Y, Wu Q, Ma S, Ma T, Shan L, Wang X, Nie Y, Ning Z, Yan L, Xiu Y, Wei F (2017). Comparative genomics reveals convergent evolution between the bamboo-eating giant and red pandas. Proc Natl Acad Sci U S A.

[3] Ledje C, Arnason U (1996). Phylogenetic relationships within caniform carnivores based on analyses of the mitochondrial 12S rRNA gene. J Mol Evol.

[4] Hu Y, Thapa A, Fan H, Ma T, Wu Q, Ma S (2020). Genomic evidence for two phylogenetic species and long-term population bottlenecks in red pandas. Sci. Adv.

[5] Wei F, Hu Y, Zhu L, Bruford MW, Zhan X, Zhang L (2012). Black and white and read all over: the past, present and future of giant panda genetics. Mol Ecol.

[6] Agnarsson I, Kuntner M, May-Collado LJ (2010). Dogs, cats, and kin: a molecular species-level phylogeny of Carnivora. Mol Phylogenet Evol.

[7] Peng R, Zeng B, Meng X, Yue B, Zhang Z, Zou F (2017). The complete mitochondrial genome and phylogenetic analysis of the giant panda (*Ailuropoda melanoleuca*). Gene.

[8] Fulton TL, Strobeck C (2017). Novel phylogeny of the raccoon family (Procyonidae: Carnivora) based on nuclear and mitochondrial DNA evidence. Mol Phylogenet Evol.

[9] Yu L, Zhang YP (2006). Phylogeny of the caniform carnivora: evidence from multiple genes. Genetica.

[10] Sato JJ, Wolsan M, Minami S, Hosoda T, Sinaga MH, Hiyama K (2009). Deciphering and dating the red panda's ancestry and early adaptive radiation of Musteloidea. Mol Phylogenet Evol.

[11] Sato JJ, Wolsan M, Suzuki H, Hosoda T, Yamaguchi Y, Hiyama K, Kobayashi M, Minami S (2006). Evidence from nuclear DNA sequences sheds light on the phylogenetic relationships of Pinnipedia: single origin with affinity to Musteloidea. Zool Sci.

[12] Nie W, Wang J, O'Brien PC, Fu B, Ying T, Ferguson-Smith MA (2002). The genome phylogeny of domestic cat, red panda and five mustelid species revealed by comparative chromosome painting and G-banding. Chromosom Res.

[13] Yu L, Luan PT, Jin W, Ryder OA, Chemnick LG, Davis HA, Zhang YP (2011). Phylogenetic utility of nuclear introns in interfamilial relationships of Caniformia (order Carnivora). Syst Biol.

[14] Delisle I, Strobeck C (2005). A phylogeny of the Caniformia (order Carnivora) based on 12 complete protein-coding mitochondrial genes. Mol Phylogenet Evol.

[15] Cserhati M, Xiao P, Guda C (2019). K-mer based motif analysis in insect species across *Anopheles*, *Drosophila* and *Glossina* genera and its application to species classification. Comput Mathe Methods Med.

[16] Heath TA, Zwickl DJ, Kim J (2008). Hillis DM taxon sampling affects inferences of macroevolutionary processes from phylogenetic trees. Syst Biol.

[17] Wood DE, Salzberg SL. Kraken: ultrafast metagenomic sequence classification using exact alignments.Genome Biol. 2014;15(3):R46. 10.1186/gb-2014-15-3-r46. 10.1186/gb-2014-15-3-r46 PMC4053813 24580807

[18] Rosen G, Garbarine E, Caseiro D, Polikar R, Sokhansanj B. Metagenome fragment classification using N-mer frequency profiles. Advances in bioinformatics. 2008;205969. 10.1155/2008/205969. 10.1155/2008/205969 PMC2777009 19956701

[19] Brady A, Salzberg SL (2009). Phymm and PhymmBL: metagenomic phylogenetic classification with interpolated Markov models. Nat Methods.

[20] Krause J, Unger T, Noçon A, Malaspinas AS, Kolokotronis SO, Stiller M, Soibelzon L, Spriggs H, Dear PH, Briggs AW, Bray SCE, O'Brien SJ, Rabeder G, Matheus P, Cooper A, Slatkin M, Pääbo S, Hofreiter M (2008). Mitochondrial genomes reveal an explosive radiation of extinct and extant bears near the Miocene-Pliocene boundary. BMC Evol Biol.

[21] Yu L, Li YW, Ryder OA, Zhang YP (2007). Analysis of complete mitochondrial genome sequences increases phylogenetic resolution of bears (Ursidae), a mammalian family that experienced rapid speciation. BMC Evol Biol.

[22] Fulton TL, Strobeck C (2016). Molecular phylogeny of the Arctoidea (Carnivora): effect of missing data on supertree and supermatrix analyses of multiple gene data sets. Mol Phylogenet Evol.

[23] Mitchener CD, Sokal RR (1956). A quantitative approach to a problem in classification. Evolution.

[24] Vinga S, Almeida J (2003). Alignment-free sequence comparison-a review. Bioinformatics.

[25] Pollard DA, Iyer VN, Moses AM, Eisen MB (2006). Widespread discordance of gene trees with species tree in Drosophila: evidence for incomplete lineage sorting. PLoS Genet.

[26] Yang K, Zhang L (2008). Performance comparison between k-tuple distance and four model-based distances in phylogenetic tree reconstruction. Nucleic Acids Res.

[27] Saitou N, Nei M (1987). The neighbor-joining method: a new method for reconstructing phylogenetic trees. Mol Biol Evol.

[28] Tamura K, Nei M (1993). Estimation of the number of nucleotide substitutions in the control region of mitochondrial DNA in humans and chimpanzees. Mol Biol Evol.

[29] Kumar S, Stecher G, Li M, Knyaz C, Tamura K (2018). MEGA X: molecular evolutionary genetics analysis across computing platforms. Mol Biol Evol.

[30] Edgar RC (2004). MUSCLE: multiple sequence alignment with high accuracy and high throughput. Nucleic Acids Res.

[31] Hall TA (1999). BioEdit: a user-friendly biological sequence alignment editor and analysis program for Windows 95/98/NT. Nucl Acids Symp Ser.

