# Supplementary figures and images for: A tail of two pandas— whole genome k-mer signature analysis of the red panda (Ailurus fulgens) and the Giant panda (Ailuropoda melanoleuca)
Source: BMC Genomics. 2021 Apr 1;22:228. doi: 10.1186/s12864-021-07531-3 (PMC8015091; doi:10.1186/s12864-021-07531-3)

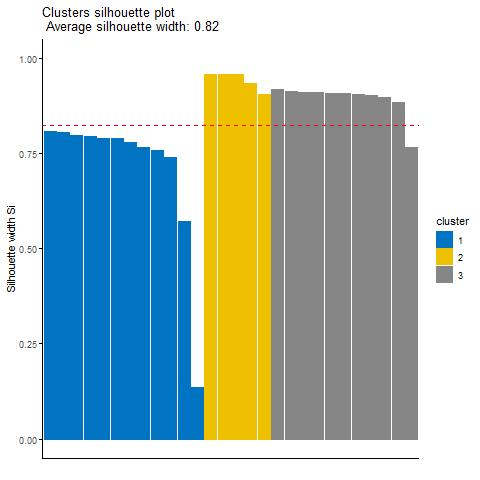

Supplement: Supplementary file 3 — Additional file 3: Figure S1. Silhouette plot for three clusters from the WGKS analysis. The average silhouette width is 0.82. [file 12864_2021_7531_MOESM3_ESM.tiff]

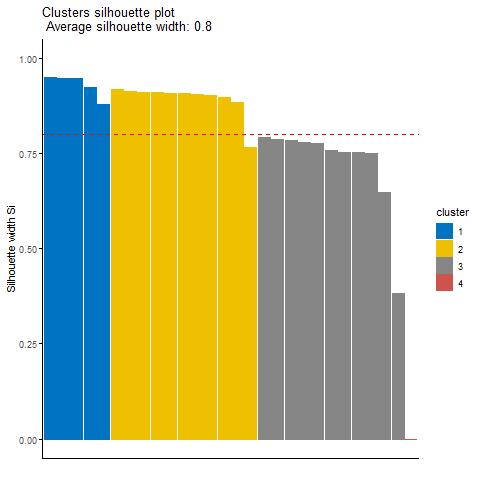

Supplement: Supplementary file 4 — Additional file 4: Figure S2. Silhouette plot for four clusters from the WGKS analysis. The average silhouette width is 0.8. [file 12864_2021_7531_MOESM4_ESM.tiff]

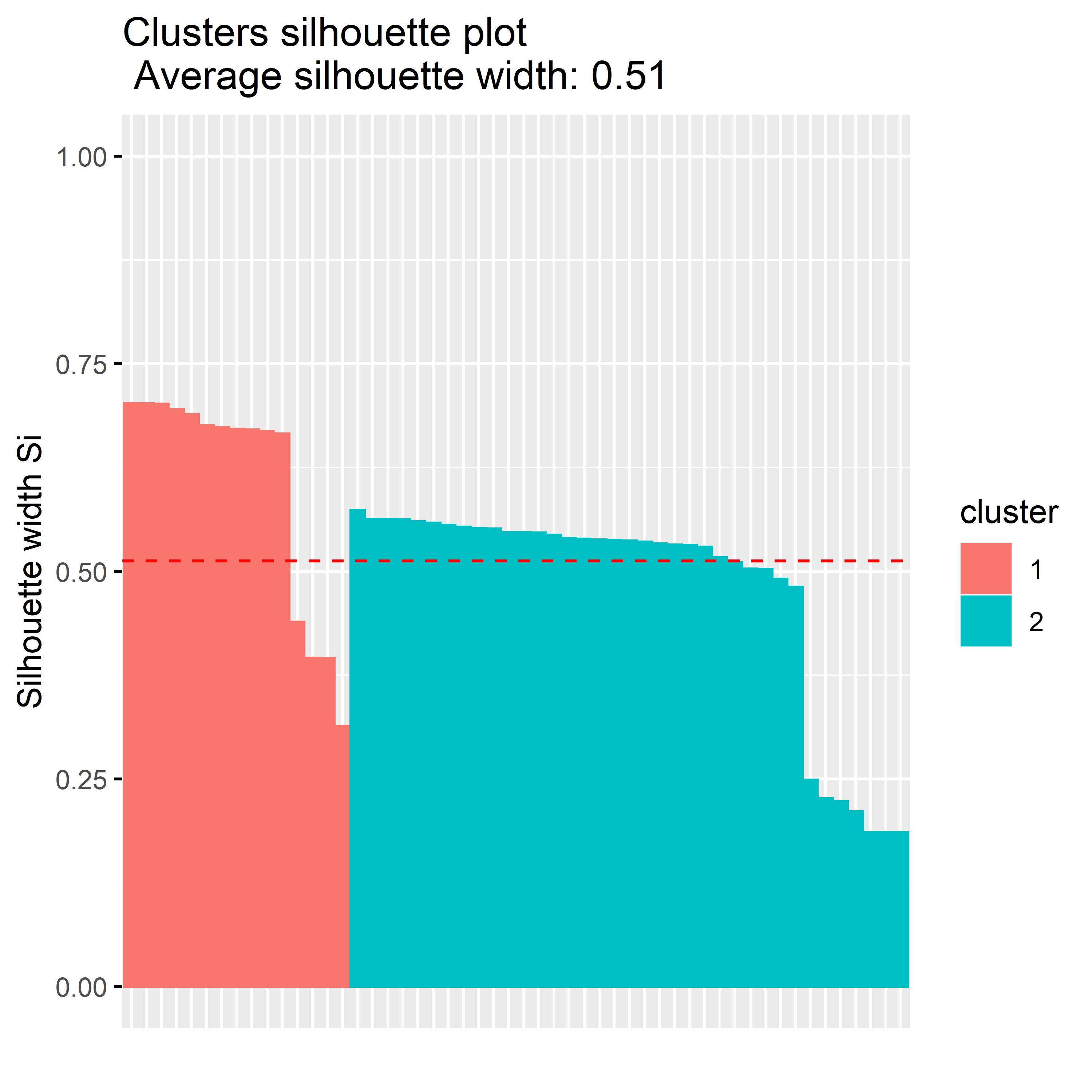

Supplement: Supplementary file 5 — Additional file 5: Figure S3. Plot showing the mean silhouette width according to the number of clusters for the mitochondrial data, based on the ‘silhouette’ method. The maximum average silhouette width is 0.51 for two clusters. [file 12864_2021_7531_MOESM5_ESM.tiff]
